# Wie lässt sich die Eliminierung von Hepatitis B, C und D in Deutschland messen? Ergebnisse eines interdisziplinären Arbeitstreffens

**DOI:** 10.1007/s00103-020-03260-2

**Published:** 2020-12-16

**Authors:** Ruth Zimmermann, Wiebe Külper-Schiek, Gyde Steffen, Sofie Gillesberg Lassen, Viviane Bremer, Sandra Dudareva, die Hepatitis-Monitoring-Arbeitsgruppe, Claus-Thomas Bock, Tanja Charles, Sandra Ciesek, Michaela Diercke, Johannes Friesen, Miriam Gerlich, Dieter Glebe, Osamah Hamouda, Thomas Harder, Renate Heyne, Alexandra Hofmann, Carlo Kantwerk, Heiko Karcher, Achim Kautz, Christian Kollan, Uwe Koppe, Klaus Kraywinkel, Katrin Kremer, Lars E. Kroll, Tina Lohse, Benjamin Maasoumy, Binod Mahanty, Ulrich Marcus, Hrvoje Mijočević, Lukas Murajda, Martin Obermeier, Ruth Offergeld, Jördis J. Ott, Karina Preußel, Claudia Santos-Hövener, Christoph Sarrazin, Armin Schafberger, Martin Schlaud, Daniel Schmidt, Christian Schmidt, Bernd Schulte, Stefan Scholz, Frank Tacke, Regina Maria Selb, Carsten Tiemann, Jörg Timm, Florian van Bömmel, Petra von Berenberg-Gossler, Jochen Walker, Heiner Wedemeyer

**Affiliations:** 1 Abteilung für Infektionsepidemiologie, Fachgebiet 34 HIV/AIDS und andere sexuell oder durch Blut übertragene Infektionen, Robert Koch-Institut, Seestr. 10, 13353 Berlin, Deutschland; 2 Abteilung für Infektionsepidemiologie, Fachgebiet 33 Impfprävention, Robert Koch-Institut, Berlin, Deutschland; 3 Abteilung für Infektionsepidemiologie, Fachgebiet 35 Gastrointestinale Infektionen, Zoonosen und tropische Infektionen, Robert Koch-Institut, Berlin, Deutschland; 4 Charité – Universitätsmedizin Berlin, Berlin, Deutschland

**Keywords:** Indikatoren, Allgemeinbevölkerung, vulnerable Gruppen, Epidemiologie, Datenquellen, Sekundärdaten, Indicators, General population, Vulnerable groups, Epidemiology, Data sources, Secondary data

## Abstract

**Hintergrund:**

Die Weltgesundheitsorganisation (WHO) hat 2016 eine Strategie zur Eliminierung von Hepatitis-B-, -C- und -D-Virusinfektionen verfasst und Indikatoren zum Monitoring des Fortschritts definiert. Das Robert Koch-Institut hat 2019 ein interdisziplinäres Arbeitstreffen zur Verbesserung der Datenlage veranstaltet.

**Ziele:**

Ziele waren die Vernetzung der Akteure, die Erstellung einer Übersicht zu den in Deutschland vorhandenen Datenquellen zu Hepatitis B, C und D und die Diskussion methodischer Aspekte.

**Material und Methoden:**

Die für Deutschland relevanten WHO-Indikatoren wurden extrahiert und es wurde bestimmt, wie diese anhand vorliegender Daten konstruiert werden können. Bei dem Arbeitstreffen mit AkteurInnen aus dem öffentlichen Gesundheitsdienst, aus Kliniken, Laboren, von Krankenkassen, Forschungsinstituten, Datenhaltern und Registern wurden in Arbeitsgruppen Erhebungsmethoden diskutiert, welche dazu dienen können, fehlende Daten zu ermitteln. Die Datenquellen und Daten wurden hinsichtlich Qualität, Vollständigkeit sowie praktischer Umsetzbarkeit evaluiert und priorisiert.

**Ergebnisse:**

Für die Allgemeinbevölkerung können die Indikatoren zu Prävention, Testung, Diagnose, Behandlung, Heilung, Folgeschäden und Mortalität aus Diagnose‑, Versorgungs- und Registerdaten, Daten aus Laboren und klinischen Zentren sowie einzelnen Studien konstruiert werden. Datenquellen für vulnerable Gruppen beschränken sich auf einzelne Studien zu Drogengebrauchenden, Männern, die Sex mit Männern haben, und HIV-Ko-Infizierten. Daten für MigrantInnen, Inhaftierte und SexarbeiterInnen sind kaum verfügbar; ebenso fehlen aktuelle Daten zur Krankheitslast chronischer Hepatitisinfektionen in der Allgemeinbevölkerung.

**Diskussion:**

Für alle ausgewählten Indikatoren konnten Datenquellen, ihre Besonderheiten und Limitationen identifiziert werden. Im nächsten Schritt gilt es, die entwickelten Ideen in konkrete Projekte mit einzelnen Datenhaltern umzusetzen.

**Zusatzmaterial online:**

Zusätzliche Informationen sind in der Online-Version dieses Artikels (10.1007/s00103-020-03260-2) enthalten.

## Abstract (en)

**Background:**

In 2016, the World Health Organization (WHO) released a strategy to eliminate hepatitis B, C, and D and defined indicators to monitor the progress. The Robert Koch Institute organized an interdisciplinary working meeting in 2019 to identify data sources and gaps.

**Objectives:**

The objectives were to network, to create an overview of the data sources available in Germany on hepatitis B and C, and to discuss how to construct indicators.

**Materials and methods:**

We extracted the WHO indicators relevant for Germany and determined how they can be constructed on the basis of available data. Stakeholders from public health services, clinics, laboratories, health insurance companies, research institutes, data holders, and registries attended a workshop and discussed methods of constructing the indicators for which data are lacking. Data sources and data were evaluated and prioritized with regard to their quality and completeness.

**Results:**

Indicators on prevalence, incidence, prevention, testing and diagnosis, treatment, cure, burden of sequelae, and mortality for the general population can be constructed using secondary data such as diagnosis, health service, and registry data, data from laboratories and hospitals as well as population-based studies. Data sources for vulnerable groups are limited to studies among drug users, men who have sex with men, and about HIV coinfected patients. Data for migrants, prisoners, and sex workers are largely lacking as well as data on burden of disease from chronic viral hepatitis in the general population.

**Conclusions:**

We identified data sources, their limitations, and methods for construction for all selected indicators. The next step is to convert the ideas developed into concrete projects with individual stakeholders.

## Einleitung

Infektionen durch Hepatitis-B- und -C-Viren (HBV, HCV) gehören zu den häufigsten Infektionen weltweit und können durch ihren chronischen Verlauf schwere Folgeschäden wie Leberzirrhose und Leberzellkarzinom verursachen. Sie rangieren in der globalen Todesursachenstatistik unter den 10 bedeutendsten Todesursachen weltweit [1–4]. Das Hepatitis-D-Virus (HDV) benötigt HBV zur Replikation. Eine HBV-HDV-Co-Infektion verläuft häufig schwer und chronisch [5]. In Europa sind mehr Todesfälle auf chronische Virushepatitiden als auf Tuberkulose und HIV/AIDS zurückzuführen. Zwar gilt Deutschland im weltweiten Vergleich als Niedrigprävalenzland für Virushepatitis [6, 7], jedoch belegt die Einstufung von HBV und HCV bei einer Priorisierung von 127 Erregern auf die Plätze 4 und 5 ihre große Bedeutung für die nationale Surveillance in Deutschland [8]. Zudem sind vulnerable Gruppen in Deutschland in höherem Maße als die Allgemeinbevölkerung von Hepatitis B und C betroffen [9].

Im Mai 2016 hat die Weltgesundheitsorganisation (WHO) die erste Strategie mit der Vision einer Eliminierung der Virushepatitis, als Bedrohung der öffentlichen Gesundheit, bis zum Jahr 2030 verabschiedet [10]. Diese ist an die nachhaltigen Entwicklungsziele der Vereinten Nationen (*Sustainable Development Goals*) angelehnt [11]. Die Länder der europäischen WHO-Region (WHO Europa) haben im September 2016 einen entsprechenden Aktionsplan verabschiedet [12]. Mit der Strategie zur Eindämmung von HIV, Hepatitis B und C und anderen sexuell übertragbaren Infektionen („BIS 2030 – Bedarfsorientiert, Integriert, Sektorübergreifend“) zielt das Bundesministerium für Gesundheit (BMG) auf die nachhaltige Eindämmung dieser Infektionskrankheiten in Deutschland ab [13].

**Figure 1:**
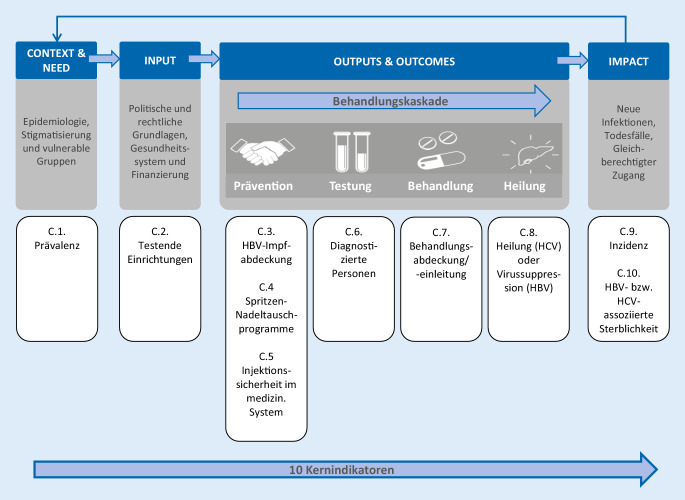

Von der WHO wurden Kern- und Zusatzindikatoren definiert, die für die Beschreibung der Ausgangssituation und die Überwachung im zeitlichen Verlauf zukünftig regelmäßig zu erheben sind [14]. Diese sind den übergeordneten Bereichen *Context and Need* (Epidemiologie), *Inputs* (System), *Outputs and Outcomes* (mit den Kategorien Prävention, Testung, Behandlung, Heilung) sowie *Impact* (Eliminierung) zugeordnet (Abb. 1). Für WHO Europa wurden einige Indikatoren angepasst bzw. ergänzt [12].

Manche Indikatoren beziehen sich auf die Gesamtbevölkerung, manche auf Bevölkerungsgruppen, die besonders vulnerabel bzw. exponiert gegenüber Virushepatitis sind (nachfolgend als vulnerable Gruppen bezeichnet). Mitgliedsstaaten sollen regelmäßig die Indikatoren an die WHO Europa und das *European Centre for Disease Prevention and Control* (ECDC) in standardisierter Form berichten [15, 16].

Für die Erhebung der Indikatoren für Deutschland können epidemiologische Daten aus verschiedenen Datenquellen genutzt werden, z. B. aus Prävalenzstudien des Robert Koch-Instituts (RKI), den Meldedaten nach Infektionsschutzgesetz (IfSG) und den Schuleingangsuntersuchungen (s. Online-Zusatzmaterial, Tab. Z‑1) unter Beachtung der jeweiligen Limitationen. Eine zusätzliche Datenressource zur Erhebung der Indikatoren ist ein Scoping-Review zur Datenlage von Hepatitis B und C in Deutschland (Hep-Epi-Projekt), welches zahlreiche Datenlücken aufdeckte, z. B. zur Inzidenz, Mortalität und zu Spätfolgen der Hepatitis B und C sowie zur sog. Behandlungskaskade [9, 17]. Eine weitere Datenquelle ist die Nationale Kohorte, eine populationsbasierte deutsche Kohortenstudie, die eine Gesamtteilnehmerzahl von 200.000 Erwachsenen anstrebt. Bei der Basisrekrutierung 2014–2019 erfolgte die Erhebung des selbstberichteten Infektionsstatus, auch für HBV und HCV [18].

Teilweise sind Daten auch in Kliniken, Laboren, Registern oder Datenkörpern der Routineversorgung vorhanden. Diese könnten bei einer Zusammenarbeit mit entsprechenden Institutionen für eine Basiserhebung genutzt werden und gegebenenfalls auch eine wiederholte Evaluation der Hepatitis-B- und Hepatitis-C-Situation in Deutschland ermöglichen.

Um zu eruieren, welche Datenquellen zukünftig für die Konstruktion der Indikatoren relevant sind, und um Ansätze für die Schließung der Datenlücken zu erarbeiten, hat das RKI ein Arbeitstreffen mit Akteuren aus verschiedenen Bereichen der Gesundheitsversorgung und -berichterstattung durchgeführt.

Das eintägige Arbeitstreffen am RKI im November 2019 hatte drei Ziele:

1. Akteure der verschiedenen Institutionen zusammenzubringen,
2. Eine Übersicht über die in Deutschland vorhandenen Datenquellen zur Hepatitis B und C zu erstellen und
3. Gemeinsam Ideen zu entwickeln, wie die von der WHO definierten und für das Arbeitstreffen als relevant erachteten Indikatoren für Deutschland generiert werden können.

Dabei wurden die vorhandenen Datenquellen auf ihre Eignung zur Erhebung der einzelnen Indikatoren evaluiert und neue Projektideen skizziert, um bestehende Datenlücken schließen zu können.

## Methoden

### Vorarbeiten durch das RKI

Von den WHO- bzw. WHO-Europa-Indikatoren wurden die für die epidemiologische Situation in Deutschland relevanten Indikatoren extrahiert und, wenn nötig, angepasst. Im nächsten Schritt wurde überprüft, ob und wie die Indikatoren anhand der vorliegenden Daten (RKI-Studien, Meldedaten und Hep-Epi-Projekt) bestimmt werden können. Für die Indikatoren, für die vorab keine geeignete Datenquelle identifiziert werden konnte, sollten auf dem Arbeitstreffen Erhebungsmodalitäten definiert werden.

### Arbeitstreffen

Im November 2019 fand ein eintägiges Arbeitstreffen am RKI mit Akteuren aus dem öffentlichen Gesundheitsdienst, aus Kliniken, Laboren, von Krankenkassen, Forschungsinstitutionen, Sekundärdaten^1^-Halter und Registern statt. VertreterInnen von klinischen Registern und Sekundärdaten-Halter stellten in Kurzvorträgen ihre Datenquellen in Bezug auf Hepatitis B und C sowie Möglichkeiten der Kooperation zur Datenerhebung und -auswertung vor. In fünf vorab definierten Arbeitsgruppen (AGs) wurden Möglichkeiten zur Generierung der einzelnen Indikatoren erarbeitet. Dazu wurde jeder AG eine zugeschnittene Indikatorenliste (Name, Definition und Konstruktion des Indikators) zur Verfügung gestellt. Einige Indikatoren wurden in mehreren AGs behandelt, um Vor- und Nachteile der jeweiligen Datenquellen zu prüfen.

Die AGs setzten sich folgendermaßen zusammen:

- AG1: nationale Referenzzentren für Hepatitis B, Hepatitis C, Forschungs- und Routinelabore;
- AG2: Institutionen, die Sekundärdaten (insbesondere Sozialdaten) halten oder mit diesen arbeiten: private Krankenversicherungen (PKV) und gesetzliche Krankenkassen, Zentralinstitut für die kassenärztliche Versorgung in Deutschland (Zi), Deutsches Institut für Medizinische Dokumentation und Information (DIMDI, jetzt Bundesinstitut für Arzneimittel und Medizinprodukte [BfArM]);
- AG3: klinische Register, klinische Zentren mit Hepatitisschwerpunkt;
- AG4: Zentrum für Krebsregisterdaten und Statistisches Bundesamt;
- AG5: Deutsche AIDS-Hilfe (DAH), Zentrum für interdisziplinäre Suchtforschung, Deutsche Beobachtungsstelle für Drogen und Drogensucht (DBDD), Abteilung für Epidemiologie und Gesundheitsmonitoring des RKI, Arbeitskreis AIDS niedergelassener Ärzte Berlin, Deutsche Leberhilfe e. V.

Die AG-Mitglieder vertraten verschiedene Datenquellen. Tab. Z‑1 im Online-Zusatzmaterial fasst diese und weitere Datenquellen jeweils mit ihren Besonderheiten und Limitationen zusammen.

Im Abschlussplenum wurden die Ergebnisse der AGs vorgestellt und diskutiert. Im Nachgang wurden sie durch das RKI ergänzt sowie hinsichtlich der Güte der Erhebungsmethode und der praktischen Umsetzbarkeit evaluiert und priorisiert. Für jeden Indikator wurde geprüft, welche Datenquelle welche Daten in welcher Qualität liefern kann.

### Darstellung der Ergebnisse

Die Ergebnisse der Diskussionen werden entsprechend den in Abb. 1 genannten übergeordneten Bereichen gegliedert für die Allgemeinbevölkerung dargestellt. Die Kern- (*Core*, C.) und Zusatzindikatoren (*Additional*, A.) wurden inkl. ihrer Definition und Konstruktion tabellarisch zusammengefasst und die Datenquellen nach Vollständigkeit und Verfügbarkeit in 4 Kategorien eingeteilt. Die im Nachgang identifizierte Hauptdatenquelle ist dargestellt.

Zur Vermeidung von Redundanzen werden die Datenquellen, mit denen Indikatoren für verschiedene vulnerable Gruppen generiert werden können, im Online-Zusatzmaterial (Tab. Z‑2) vorgestellt.

## Ergebnisse

### Indikatoren im Bereich* Context and Need* (Epidemiologie)

Die Kernindikatoren dieses Bereichs umfassen die „Prävalenz von chronischer HBV- und HCV-Infektionen“ (C.1.a, C.1.b) und den Zusatzindikator „Hepatitis-D-Co-Infektionen bei Personen mit chronischer HBV-Infektion“ (A.1) (Tab. 1).

**Table 1:** 

Bereich	Indikator				Daten aus Laboren	Daten aus klinischen Zentren	Sekundärdaten	Daten aus epidemiologischen Studien, Surveillance	Hauptdatenquelle (s. Tab. Z‑1)
	Nummer	Name	Definition	Konstruktion					
*Context and Need* *(Epidemiologie)*	*C.1.a*	*Prävalenz chronischer HBV-Infektionen*	Anzahl und Anteil Personen mit chronischer HBV-Infektion (HBsAg positiv)	Z: Anzahl HBsAg-positiver Personen (=mit chronischer HBV-Infektion)	+	+	+	++	Erwachsene: DEGS- [19], gern-Studie Kinder: KiGGS-Studie [20]
				N: Gesamtpopulation					
	*C.1.b*	*Prävalenz chronischer HCV-Infektionen*	Anzahl und Anteil Personen mit chronischer HCV-Infektion (HCV-RNA oder HCV-Ag positiv)	Z: Anzahl HCV-RNA- oder HCV-Ag-positiver Personen	−	+	+	++	Erwachsene: DEGS- [19], gern-Studie Kinder: KiGGS-Studie [20]
				N: Gesamtpopulation					
	*A.1*	*Hepatitis-D-Co-Infektionen bei Personen mit chronischer HBV-Infektion*	Anzahl und Anteil HDV-infizierter Personen an allen HBV-infizierten Personen	Z: Anzahl HBsAg-positiver und anti-HDV-positiver Personen	+	++	−	−	Projekt zur HDV-Prävalenz bei HBsAg-positiven PatientInnen in Zentren der Deutschen Leberstiftung
				N: Anzahl HBsAg-positiver Personen					

− nicht favorisiert, + mögliche Datenquelle, ++ Hauptdatenquelle

*C* Kernindikator, *A* Zusatzindikator, *Z* Zähler, *N* Nenner, *HBV* Hepatitis-B-Virus, *HCV* Hepatitis-C-Virus, *HDV* Hepatitis-D-Virus, *HBsAg* Hepatitis B surface Antigen, *RNA* Ribonukleinsäure, *Ag* Antigen

Für die Bestimmung der Prävalenz von Hepatitis B und C in der Allgemeinbevölkerung werden regelmäßig die Daten von bevölkerungsbasierten Studien (z. B. Deutsche Erwachsenen- und Kindergesundheitsstudie (DEGS bzw. KiGGS) [19–21]) genutzt. Für die Basiserhebung der Indikatoren C.1.a und C.1.b sind diese Daten aufgrund der Verfügbarkeit und der repräsentativen Stichprobe am geeignetsten. Für Folgeerhebungen können zukünftig durchgeführte Studien (z. B. die Gesundheits- und Ernährungsstudie in Deutschland, gern-Studie) verwendet werden. Über die Versichertennummer gesetzlich Versicherter wäre anhand von Labordaten auch eine Bestimmung der HBsAg-Positivenrate unter den getesteten Personen möglich. Zudem können Daten aus der Blutspende-Surveillance für die Erhebung der Basis- und zukünftigen Prävalenz (HBV- und HCV-Prävalenz bei Erstspendern) in der nichtexponierten Allgemeinbevölkerung näherungsweise herangezogen werden. Die Prävalenz von Hepatitis D (HDV) unter HBV-Infizierten (A.1) könnte mittels Fragebögen in größeren Leberzentren der Deutschen Leberstiftung geschätzt werden.

Alternativ können für die genannten Indikatoren auch Sekundärdaten oder Daten aus klinischen Zentren (vor allem für die Erhebung der Prävalenz in bestimmten Subpopulationen) genutzt werden. Da Sekundärdaten nur diagnostizierte Fälle abbilden, werden dabei eigentlich Diagnoseprävalenzen erhoben.

### Indikatoren im Bereich* Input* (System)

Der Bereich *Input* umfasst den Kernindikator „Testende Einrichtungen“ (C.2). Dieser Indikator ist in Deutschland für vulnerable Gruppen relevant und wurde daher entsprechend modifiziert („Anzahl an Einrichtungen, die eine Testung in Risikogruppen durchführen können“). Er wird unter „Vulnerable Gruppen“ beschrieben (Online-Zusatzmaterial, Tab. Z‑2).

### Indikatoren im Bereich* Outputs and Outcomes*

Im Bereich *Outputs and Outcomes* sind Kernindikatoren in den Kategorien „Prävention“, „Testung“, „Behandlung“ und „Heilung“ zusammengefasst, die die Behandlungskaskade der Hepatitis B und C abbilden (Abb. 1). Vier der sechs Kernindikatoren (C.3, C.6, C.7, C.8) wurden als relevant für Deutschland eingestuft. Sie werden im Folgenden beschrieben.

#### Indikatoren der Kategorie „Prävention“

In die Kategorie „Prävention“ fallen Indikatoren zur Umsetzung der Hepatitis-B-Impfung, zur Prävention der Mutter-Kind-Übertragung von HBV, zur Sicherheit von Blutprodukten sowie schadensminimierende Maßnahmen bei Drogengebrauchenden (Tab. 2; s. auch Kapitel „Vulnerable Gruppen“).

**Table 2:** 

Bereich – Kategorie		Indikator				Daten aus Laboren	Daten aus klinischen Zentren	Sekundärdaten	Daten aus epidemiologischen Studien, Surveillance	Hauptdatenquelle (s. Tab. Z‑1)
		Nummer	Name	Definition	Konstruktion					
*Outputs and Outcomes*	*Umsetzung der Hepatitis-B-Impfung*	*C.3.b*	*Impfrate für die 3. HBV-Impfstoffdosis bzw. Rate vollständiger und zeitgerecht durchgeführter Impfserien bei Kindern*	Abdeckung der 3. HBV-Impfstoffdosis bei Kindern bzw. Rate an nach nationalen Richtlinien vollständig und zeitgerecht geimpften Kindern	Z: Anzahl Kinder, die nach nationalen Richtlinien vollständig und zeitgerecht geimpft sind	−	−	++	+	Z: RKI-Impf-Surveillance [23], Daten des WIP, KiGGS-Studie [20]
					N: Anzahl Kinder in Deutschland					N: Destatis
		*A.16*	*Hepatitis-B‑Impfquoten bei Gesundheitspersonal*	Abdeckung der Hepatitis-B-Impfung bei Gesundheitspersonal	Z: HBV-geimpftes Gesundheitspersonal	−	−	−	++	OKaPII [24, 25]
					N: Anzahl Gesundheitspersonal					
		*Deutschland*	*Anteil geimpfter Personen in vulnerablen Gruppen*	Anzahl und Anteil geimpfter Personen in vulnerablen Gruppen	Z: Anzahl geimpfter Personen in vulnerablen Gruppen	−	−	+	++	Daten aus Studien bei vulnerablen Gruppen, Tab. Z‑2
					N: Größe der vulnerablen Gruppe					
	*Prävention – Mutter-Kind-Übertragung*	*WHO Europa*	*HBsAg-Screening bei Schwangeren*	HBsAg-Screening bei Schwangeren	Z: Anzahl auf HBsAg gescreente Schwangere	+	−	++	−	Z: Abrechnungsdaten (Zi/InGef/Techniker Krankenkasse)
					N: Anzahl aller Schwangeren					N: Destatis (Anzahl der Frauen mit Lebendgeburten zuzüglich der Totgeburten)
		*C.3.a*	*HBV-PEP bei Neugeborenen HBV-infizierter Mütter*	Anteil Neugeborener von HBV-infizierten Müttern, die HBV-PEP erhalten haben	Z: Anzahl Kinder HBsAg-positiver Mütter, die HBV-PEP erhalten haben	−	+	++	+	Abrechnungsdaten der Techniker Krankenkasse
					N: Anzahl Kinder HBsAg-positiver Mütter					
		*WHO Europa*	*0,5* *% HBsAg-Prävalenz in geimpften Kohorten*	Anteil HBV-Infizierter in Alterskohorten, die nach Einführung der HBV-Impfung geboren sind	Z: Anzahl HBsAg-positiver Kinder in Alterskohorten, die nach Einführung der HBV-Impfung geboren sind	−	−	+	++	HBsAg-Prävalenz aus KiGGS [20]
					N: Gesamtzahl Kinder in Alterskohorten, die nach Einführung von HBV-Impfung geboren sind					
	*Prävention – Blutsicherheit*	*A.17*	*Erfüllte Sicherheitsstandards bei Bluttransfusionen in Einrichtungen*	Anteil Gesundheitseinrichtungen, die Bluttransfusionen gemäß den Sicherheitsstandards durchführen	n. z.	−	−	−	++	Gesetzlich im Transfusionsgesetz durch Richtlinie Hämotherapie verankert [27, 28]. Institutionale Zuständigkeit der Hämovigilanz beim Paul-Ehrlich-Institut
		*A.18*	*Abdeckung des Screenings von Blutspenden auf HBV und HCV*	Anteil Blutproben, die auf blutübertragene Erkrankungen überprüft werden	n. z.	−	−	−	−	Gesetzliche Vorgabe; dadurch, dass Blutprodukte zugelassene Arzneimittel sind, wird dieser Indikator voll erfüllt. Meldepflicht zu Ergebnissen der Blutspendetestung [27, 28]
	*Prävention – Schadensminimierung*	*C.5*	*Spritzen-Nadel-Tauschprogramme*	Zahl der pro Jahr und Person abgegebenen sterilen Spritzen und Nadeln im Rahmen von Spritzentauschprogrammen	Z: Anzahl an sterilen Nadeln und Spritzen, die in den letzten 12 Monaten durch Nadel-Spritzen-Programme verteilt wurden	−	−	−	++	Projekt der Deutschen Beobachtungsstelle für Drogen und Drogensucht in Kooperation mit RKI und Deutscher AIDS-Hilfe
					N: Anzahl Menschen, die sich Drogen injizieren					N: Proxy: Anzahl Opioidabhängiger in Deutschland [47]
		*A.24*	*Abdeckung der Opioidsubstitution (OST)*	Anteil Drogengebrauchender, die OST erhalten	Z: Anzahl Personen unter OST	−	−	−	++	Jährlicher Substitutionsbericht [46]
					N: Anzahl Opioidabhängiger in Deutschland					Anzahl Opioidabhängiger in Deutschland [47]

− nicht favorisiert, + mögliche Datenquelle, ++ Hauptdatenquelle

*C* Kernindikator, *A* Zusatzindikator, *Z* Zähler, *N* Nenner, *n. z.* nicht zutreffend

##### Umsetzung der Hepatitis-B-Impfung.

Neben dem Kernindikator zu Impfabdeckung „Impfrate für die 3. HBV-Impfstoffdosis bzw. Rate vollständiger und zeitgerecht durchgeführter Impfserien bei Kindern“ (C.3.b), fallen die Indikatoren „Hepatitis-B-Impfquoten bei Gesundheitspersonal“ (A.16) sowie „Anteil geimpfter Personen in vulnerablen Gruppen“, für die eine HBV-Impfung in Deutschland empfohlen ist, in diese Kategorie.

Die Impfrate für die 3. HBV-Impfstoffdosis bei Kindern ist als Rate an vollständig und zeitgerecht durchgeführten Impfserien (gemäß nationaler Richtlinie) zu verstehen. Diese Daten sind über jährlich durchgeführte Schuleingangsuntersuchungen verfügbar [22]. Alternativ können auch Daten der RKI-Impf-Surveillance zu gesetzlich Krankenversicherten (GK-Versicherte; [23]) und ergänzend Daten des Wissenschaftlichen Instituts der PKV (WIP) genutzt werden sowie die KiGGS-Studiendaten.

Daten zur Impfabdeckung bei Gesundheitspersonal (A.16) wurden bereits aus lokalen und überregionalen Studien für die Vergangenheit im Hep-Epi-Projekt identifiziert. Zudem erhebt das RKI seit 2018 anhand einer „*O*nline-Befragung von *K*rankenh*a*us-*P*ersonal zur *I*nfluenza-*I*mpfung“ (OKaPII-Projekt) regelmäßig den Impfstatus zu beruflich indizierten Impfungen bei Krankenhauspersonal [24]. Im Jahr 2019 wurden auch Angaben zur Hepatitis-B-Impfung erhoben [25].

##### Prävention der Mutter-Kind-Übertragung.

Der Indikator „HBV-Postexpositionsprophylaxe (PEP) bei Neugeborenen HBV-infizierter Mütter“ (C.3.a) wird aktuell im Rahmen eines RKI-Projektes zusammen mit der Techniker Krankenkasse (TK) anhand von TK-Abrechnungsdaten generiert. Da die HBV-PEP bei Neugeborenen mit einer eigenen EBM-Ziffer abgerechnet wird, könnte dieser Indikator auch aus Zi-Daten erhoben werden. Klinische Daten könnten außerdem im Rahmen einer Kooperation zwischen Hepatologen und Gynäkologen direkt aus Kliniken (etwa im Rahmen eines klinischen Sentinels) generiert werden. Inwieweit die Daten der Qualitätskontrolle und -sicherung des Instituts für Qualität und Wirtschaftlichkeit im Gesundheitswesen (IQWiG; sog. Perinataldatensatz) für eine derartige Erhebung zugänglich und sinnvoll sind, ist noch unklar.

Der Indikator „Abdeckung des HBsAg-Screenings bei Schwangeren“ wurde in der Vergangenheit bereits aus Krankenkassenabrechnungsdaten, die dem Institut für angewandte Gesundheitsforschung Berlin (InGef) vorliegen, erarbeitet [26] und wird aktuell im Rahmen des RKI-Projektes zusammen mit der TK anhand von Abrechnungsdaten generiert. Für eine regelmäßige Erfassung stünden auch Abrechnungsdaten des Zi zur Verfügung, die ggf. durch Daten des WIP ergänzt werden könnten.

Der WHO Europa-Indikator „0,5 % HBsAg-Prävalenz in geimpften Kohorten“ fällt in diesen Bereich, als Maß für die erfolgreiche Prävention von Mutter-zu-Kind-Übertragung von Hepatitis B und für die Durchimpfung in Kinderkohorten. Die Information hierzu könnte aus Seroprävalenzstudien in den Alterskohorten, die nach Einführung der HBV-Impfung und Erreichung einen guten HBV-Impfabdeckung geboren sind, kommen. Dafür können für Deutschland KiGGS-Studiendaten benutzt werden.

##### Blutsicherheit.

Die Indikatoren zur Blutsicherheit „Erfüllte Sicherheitsstandards bei Bluttransfusionen in Einrichtungen“ (A.17) und „Abdeckung des Screenings von Blutspenden auf HBV und HCV“ (A.18) sind in Deutschland im Transfusionsgesetz gesetzlich geregelt und werden zu 100 % umgesetzt. Die Hämovigilanz fällt in die Zuständigkeit des Paul-Ehrlich-Instituts. Daten der Blutspende-Surveillance aus dem Screening von Blutspenden auf Infektionen werden kontinuierlich am RKI erhoben und ausgewertet [27, 28].

#### Indikatoren der Kategorie „Testung“

Für den Indikator „HBV-/HCV-diagnostizierte Personen“ (C.6) sind prinzipiell zwei verschiedene Erhebungsmethoden denkbar: Aus den Meldedaten nach Infektionsschutzgesetz (IfSG) kann die Anzahl der positiv getesteten Personen ermittelt werden ([29–32]; Tab. 3). Auch wäre die Generierung des Zählers über Abrechnungsdaten von Diagnosen aus Versorgungsdaten der GKV nach der Datentransparenzverordnung (DaTraV), Daten des Zi oder einzelner Krankenkassen (z. B. InGef) möglich. Als Nenner kann die Zahl der mit HBV/HCV lebenden Personen modelliert werden. Eine Hochrechnung wurde vom RKI für das Jahr 2013 für HBV und HCV erstellt – jedoch ist keine aktuellere Hochrechnung verfügbar [33].

**Table 3:** 

Bereich – Kategorie		Indikator				Daten aus Laboren	Daten aus klinischen Zentren	Sekundärdaten	Daten aus epidemiologischen Studien, Surveillance	Hauptdatenquelle (s. Tab. Z‑1)
		Nummer	Name	Definition	Konstruktion					
*Outputs and Outcomes*	*Testung*	*C.6*	*HBV-/HCV-diagnostizierte Personen*	Anteil Personen mit chronischer HBV-/HCV-Infektion, deren HBV-/HCV-Infektion diagnostiziert wurde	Z: Anzahl Personen mit diagnostizierter HBV-/HCV-Infektion	−	+	++	++	Z: Abrechnungsdaten von Diagnosen aus Versorgungsdaten der GKV (z. B. DaTraV/Zi) *alternativ* Meldedaten nach IfSG [29–32]
					N: geschätzte Anzahl Personen mit HBV-/HCV-Infektion					N: RKI-Hochrechnung zur Anzahl HBV- und HCV-infizierter Personen in Deutschland [33]
		*A.5*	*Testungen auf Hepatitis B*	HBV-Testungen	Z: Anzahl auf HBV getesteter Personen innerhalb eines Jahres mittels HBsAg-Tests	+	−	++	−	Z: Zi: Abrechnungsdaten zu durchgeführter Labordiagnostik
					N: Gesamtbevölkerung					N: Destatis
		*A.6*	*Testungen auf Hepatitis C*	HCV-Testungen	Z: Anzahl auf HCV-RNA getesteter Personen innerhalb eines Jahres mittels HCV-PCR- oder Anti-HCV-Tests	+	−	++	−	Z: Zi: Abrechnungsdaten zu durchgeführter Labordiagnostik
					N: Gesamtbevölkerung					N: Destatis
	*Behandlung*	*C.7.a*	*Behandlungsabdeckung Hepatitis B*	Anteil HBV-Infizierter, die aktuell unter Behandlung sind	Z: Anzahl chron. HBV-Infizierter in Behandlung	−	+	++	+	Z: DaTraV: Verknüpfung von Medikamentenverordnungs- und Diagnosedaten
					N: Anzahl chronischer HBV-Infektionen					N: RKI-Hochrechnung zu HBV-infizierten Personen in Deutschland [33]
		*C.7.b*	*Behandlungsbeginn Hepatitis C*	Anteil chronisch HCV-Infizierter, die die Behandlung in den letzten 12 Monaten begonnen haben	Z: Anzahl Personen mit chron. HCV-Infektionen, die die Behandlung begonnen haben	−	−	++	−	Z: DaTraV: Verknüpfung von Arzneimittelverordnungen und Abrechnungsdaten zu Behandlungsdauer
					N: Anzahl Personen mit diagnostizierter chron. HCV-Infektion					N: DaTraV: Abrechnungsdaten zu durchgeführter Labordiagnostik und Diagnose
		*Deutschland*	*Anteil behandlungsbedürftiger HBsAg-positiver Personen*	Anteil behandlungsbedürftiger HBsAg-positiver Personen	Z: Anzahl behandlungsbedürftiger HBsAg-positiver Personen	−	++	−	+	Z: Zentren der Deutschen Leberstiftung: Anzahl behandlungsbedürftiger Personen
					N: Anzahl HBV-infizierter Personen					N: RKI-Hochrechnung zu HBV infizierten Personen in Deutschland [33]
		*A.7*	*Anteil chronischer HCV-Infektionen mit Genotypinformation*	Anteil chronisch HCV-Infizierter mit vorliegender Genotypinformation	Z: Fälle chronischer HCV-Infektionen mit vorliegender Genotypisierung	+	+	++	−	Z: Zi/DaTraV: Abrechnungsziffern zu durchgeführter Genotypisierung
					N: Fälle chronischer HCV-Infektionen					N: Abrechnungsziffern zu HCV-Bestätigungstest (Zi/DaTraV)
	*Heilung*	*C.8.a*	*Virussuppression bei behandelten chron. HBV-infizierten Patienten*	Anteil behandelter HBV-Infizierter mit supprimierter HBV-Viruslast	Z: Anzahl chron. HBV-Infizierter unter Behandlung mit supprimierter Viruslast in den letzten 12 Monaten	−	++	−	+	Zentren der Deutschen Leberstiftung: Bestimmung negativer Hepatitis-B-PCR bei HBsAg-positiven Patienten
					N: Anzahl behandelter HBV-Infizierter					
		*C.8.b*	*Anteil geheilter chronisch HCV-Infizierter mit abgeschlossener Behandlung*	Anzahl HCV-Infizierter, die die Behandlung abgeschlossen haben und geheilt sind	Z: Anzahl Personen mit abgeschlossener HCV-Behandlung und anhaltender virologischer Response	−	++	++	−	Abrechnungsdaten von Diagnosen und Behandlung aus Versorgungsdaten der GKV (z. B. DaTraV/Zi) *alternativ* Deutsches Hepatitis-C-Register: Anzahl Personen mit abgeschlossener HCV-Behandlung und anhaltender virologischer Response
					N: Anzahl HCV-Infizierter mit Virusnachweis und abgeschlossener Behandlung					

− nicht favorisiert, + mögliche Datenquelle, ++ Hauptdatenquelle

*C* Kernindikator, *A* Zusatzindikator, *Z* Zähler, *N* Nenner

Alternativ ließe sich der Indikator auch aus Querschnittsstudien erstellen, indem positiv auf virale Hepatitis Getestete gefragt werden, ob ihnen ihre Diagnose bereits bekannt war. Auf diese Weise werden zukünftig für den Raum Frankfurt am Main Daten für verschiedene Subpopulationen im Rahmen eines Projektes des Deutschen Zentrums für Infektionsforschung (DZIF) generiert werden. Weitere Projekte (derzeit laufendes Check-up-2-Projekt der Universitätsklinik Leipzig in Hamburg und Schleswig-Holstein sowie ein Projekt des Leberzentrums am Checkpoint Berlin) könnten ergänzende Daten liefern.

Die Zusatzindikatoren „Testungen auf Hepatitis B bzw. Hepatitis C“ (A.5, A.6) messen die Anzahl der auf HBsAg (A.5) bzw. auf HCV-RNA (A.6) getesteten Personen. Diese Daten könnten über Labore (Anzahl durchgeführter Testungen) generiert werden oder aus Versorgungsdaten der GKV (DaTraV, Daten des Zi/der Krankenkassen, z. B. InGef).

#### Indikatoren der Kategorie „Behandlung“

Die Indikatoren C.7.a und C.7.b beschreiben jeweils den Anteil der Behandelten unter den chronisch HBV- bzw. HCV-Infizierten in einem bestimmten Zeitraum (z. B. jährlich; Tab. 3). Für HCV (C.7.b) wird die Zahl der an GK-Versicherte abgegebenen Medikamentenpackungen regelmäßig durch das RKI aus Apothekenabrechnungsdaten generiert und daraus auf die Zahl der behandelten Personen hochgerechnet [34, 35].

Der Anteil der HBV-Behandelten (C.7.a) wird derzeit in einem RKI-Projekt aus Apothekenabrechnungsdaten bestimmt. Dafür wird für die Bildung des Indikators C.7.a die Anzahl der behandlungsbedürftigen HBV-Infizierten benötigt, welche über die Zentren der Deutschen Leberstiftung erhoben werden könnte. Zusätzlich muss vorab der Anteil der nicht dauerhaft Behandelten bestimmt werden. Nach Angaben der teilnehmenden KlinikerInnen beschränkt sich eine Nichtdauertherapie bei HBV auf Schwangere und Immunsupprimierte [36], diese Daten könnten ebenfalls über Zentren der Deutschen Leberstiftung abgefragt werden. Alternativ könnten die Indikatoren C.7.a und b auch aus DaTrav-Daten unter Zusammenführung von Diagnose- und Medikamentenverordnungsdaten generiert werden.

Für den Indikator „Anteil behandlungsbedürftiger HBsAg-positiver Personen in Deutschland“ könnte eine Studie in klinischen Zentren durchgeführt werden. Der dort erhobene Anteil kann entweder direkt genutzt oder auch hochgerechnet werden auf die Gesamtzahl aller HBsAg-positiven Personen in Deutschland.

Der Indikator „Anteil chronischer HCV-Infektionen mit Genotypinformation“ (A.7) kann anhand von Sekundärdaten erhoben werden, da die durchgeführte HCV-Genotypisierung in einer eigenen Abrechnungsziffer abgebildet wird. Alternativ könnte aus Labordaten die Anzahl positiver HCV-Tests sowie die Anzahl durchgeführter Genotypisierungen erhoben werden. Allerdings liegen den Laboren in der Regel keine Daten zum Status der HCV-Erkrankung (akut versus chronisch) vor.

#### Indikatoren der Kategorie „Heilung“ (HBV-Suppression und HCV-Heilung)

Der Indikator „Virussuppression bei behandelten chronisch HBV-infizierten Personen“ (C.8.a) kann anhand von Daten aus klinischen Zentren zur Anzahl Behandelter und der Anzahl HBsAg-positiver Patienten mit negativer Hepatitis-B‑PCR generiert werden (Tab. 3). Für die Erhebung des „Anteils geheilter chronisch HCV-Infizierter mit abgeschlossener Behandlung“ (C.8.b) können sowohl Versorgungsdaten der GKV mittels Verknüpfung von Diagnose‑, Behandlungs- und ggf. Laborabrechnungsdaten verwendet werden als auch klinische Daten des Deutschen Hepatitis-C-Registers. Unter der Annahme, dass ca. 95 % der HCV-Behandelten auch geheilt werden, wäre auch eine Schätzung anhand von Apothekenabrechnungsdaten denkbar, aus welchen der Indikator für die Gesamtbevölkerung konstruiert werden könnte.

### Indikatoren im Bereich „Impact“

Der Bereich *Impact* umfasst die Inzidenz und Mortalität von Hepatitis B und C (Tab. 4).

**Table 4:** 

Bereich – Kategorie		Indikator				Daten aus Laboren	Daten aus klinischen Zentren	Sekundärdaten	Daten aus epidemiologischen Studien, Surveillance	Hauptdatenquelle (s. Tab. Z‑1)
		Nummer	Name	Definition	Konstruktion					
*Impact*	*Inzidenz*	*C.9.a*^*a*^*/C.9.b*	*Rate an HBV-/HCV-Neuinfektionen*	Anzahl und Rate neu identifizierter HBV-/HCV-Infektionen/Jahr	Z: Anzahl HBV-/HCV-Neuinfektionen pro Jahr	−	−	++	−	Z: DaTraV: Abrechnungsdaten von Diagnosen
					N: Gesamtbevölkerung minus Personen mit Hepatitisdiagnose					N: Gesamtbevölkerung ohne Anteil von Personen mit Hepatitisdiagnose aus RKI-Hochrechnung zu HBV-infizierten Personen in Deutschland [33]
		*A.26*^*a*^	*Inzidenz des hepatozellulären Karzinoms (HCC)*	Neuerkrankungen an HCC	Z: Anzahl neu diagnostizierter HCC	−	+	++	−	Z: Krebsregisterdaten
					N: Gesamtbevölkerung					N: Destatis
	*Mortalität und Folgeschäden*	*C.10*	*HBV-/HCV-bedingte Todesfälle*	Todesfälle an HBV-/HCV-assoziierten Erkrankungen: HCC, Zirrhose, chronischer Lebererkrankung (CLD)	Anzahl an Todesfällen, die auf eine HBV-/HCV-Infektion zurückzuführen sind: – HCC-Todesfälle multipliziert mit Anteil HBV-/HCV-bedingter HCC-Fälle – Todesfälle an CLD multipliziert mit Anteil HBV-/HCV-bedingter CLD – Todesfälle an Leberzirrhose multipliziert mit Anteil HBV-/HCV-bedingter Leberzirrhosefälle	+	+	++	−	Todesursachenstatistik in Kombination mit *Attributable Fraction* (AF) (jeweiliger HBV/HCV-bedingter Anteil)
										*AF für HCC*: Krebsregisterdaten: wenn zusätzliches Lebermodul implementiert werden könnte *alternativ:* DaTraV: HBV-/HCV-Diagnose + HCC-Diagnose
										*AF für Leberzirrhose*: DaTraV: HBV-/HCV-Diagnose + Leberzirrhosediagnose
		*WHO Europa*	*Spätdiagnose (Endstadium der Lebererkrankung bei HBV-/HCV-Erstdiagnose)*	Spätdiagnosen: Endstadium einer Lebererkrankung bei erstmaliger Diagnose einer HBV-/HCV-Infektion	Z: diagnostizierte HBV-/HCV-Fälle mit dekompensierter Leberzirrhose oder HCC	+	+	++	−	Z: DaTraV: Abrechnungsdaten zu HBV-/HCV-Diagnose + HCC-/Leberzirrhosediagnose
					N: Anzahl diagnostizierter HBV-/HCV-Infektionen					N: DaTraV: Abrechnungsdaten von Diagnose

− nicht favorisiert, + mögliche Datenquelle, ++ Hauptdatenquelle

*C* Kernindikator, *A* Zusatzindikator, *Z* Zähler, *N* Nenner

^a^Indikator für Deutschland adaptiert

Die Inzidenzindikatoren C.9.a und C.9.b können anhand von DaTraV-Daten erhoben werden. Alternativ kann der Indikator anhand der gemeldeten HBV‑/HCV-Fälle pro Jahr geschätzt werden. Die Inzidenz des hepatozellulären Karzinoms (HCC; A.26, adaptiert) kann durch Daten des Deutschen Krebsregisters erhoben werden.

Der Kernindikator „HBV- bzw. HCV-bedingte Todesfälle“ (C.10) umfasst HBV-/HCV-assoziierte Todesfälle, die direkt mit der HBV-/HCV-Infektion assoziiert sind oder durch HBV-/HCV-assoziierte Folgeerkrankungen wie Leberzirrhose, HCC oder andere chronische Lebererkrankungen (engl. *Chronic Liver Disease* [CLD]) bedingt sind. Bei der Erfassung der Sterbefälle an HBV‑/HCV-bedingten Folgeerkrankungen ist zu beachten, dass diese Erkrankungen auch durch andere Ursachen wie Alkoholabusus, metabolisches Syndrom (nichtalkoholische Fettleber), Autoimmunerkrankungen etc. hervorgerufen werden können. Um den Indikator generieren zu können, bedarf es daher nicht nur der Anzahl an Todesfällen nach genannten Folgeerkrankungen, sondern auch des Anteils, der auf HBV‑/HCV-Infektionen zurückzuführen ist (engl. *Attributable Fraction* [AF]).

Der Anteil der direkt HBV-/HCV-assoziierten Todesfälle sowie die Gesamtzahl von Todesfällen an HCC, Leberzirrhose oder CLD kann durch die Todesursachenstatistik abgebildet werden. Zusätzlich können diese Parameter für den stationären Bereich anhand der Krankenhausdiagnosestatistik generiert werden. Die HCC-Todesfälle können vergleichend auch mittels der Daten des Deutschen Krebsregisters erhoben werden. Die HCC-Ursache ist aktuell zwar nicht in den Krebsregisterdaten abgebildet, könnte aber über ein ergänzendes „Lebermodul“ erfasst werden, sodass dann die AF für HCC generiert werden kann. Die AF für HCC, Leberzirrhose oder CLD kann auch anhand von DaTraV-Daten erfolgen (Verknüpfung der HBV-/HCV-Diagnose mit der Diagnose HCC, Leberzirrhose oder CLD). Des Weiteren sind im Transplantationsregister ursächliche Erkrankungen für eine transplantationsbedürftige Lebererkrankung hinterlegt. Das Institut für angewandte Qualitätsförderung und Forschung im Gesundheitswesen (aQua-Institut) stellt im Rahmen der sektorenübergreifenden Qualitätssicherung auf Anfrage Daten zu Lebertransplantationen zur Verfügung.

In der AG4 (Mortalitätsdaten) wurde auch folgender innovativer Ansatz zur Schätzung der AF bei Leberzirrhose diskutiert: Anhand des APRI-Scores (Aspartat-Aminotransferase/Thrombozyten-Ratio-Index) kann durch Bestimmung dieser Laborparameter zusammen mit einem positiven HBV‑/HCV-Testergebnis auf das Vorliegen einer Leberzirrhose geschlossen werden [37]. Derart könnte auch die Anzahl spät diagnostizierter HBV-/HCV-Infektionen (WHO Europa-Indikator) mit bereits vorliegenden Folgeerkrankungen wie Leberzirrhose generiert werden. Dieser Indikator könnte auch anhand der DaTraV-Daten erhoben werden. Für HCV könnten zudem Daten des Deutschen Hepatitis-C-Registers verwendet werden, die auf eine bereits vorliegende CLD bei HCV-Erstdiagnose hin untersucht würden.

### Vulnerable Gruppen

Die verfügbaren Datenquellen zu vulnerablen Gruppen sind in Tab. Z‑1 und die Indikatoren, die für die jeweiligen Gruppen zu generieren sind, in Tab. Z‑2 (Online-Zusatzmaterial) beschrieben.

Konkret stehen für Personen, die sich Drogen injizieren (PWID) Baseline-Daten aus der DRUCK-Studie (2011–2014) zur Verfügung [38–40]. Zukünftig können voraussichtlich Daten für diese Gruppe aus einem Monitoringsystem bei Drogengebrauchenden, dessen Pilotierung für 2020 geplant ist, genutzt werden (DRUCK 2.0). Für Männer, die Sex mit Männern haben (MSM), stehen Ergebnisse der europäischen MSM-Internetsurveys (EMIS) 2010 und 2017 [41, 42] zur Verfügung. Daten zu HIV-Ko-Infizierten finden sich in Publikationen zur HIV-1-Serokonverterstudie des RKI [43, 44]. Außerdem können Abfragen über die Zentren erfolgen, die in der Deutschen Arbeitsgemeinschaft niedergelassener Ärzte in der Versorgung HIV-Infizierter e. V. (dagnä e. V.) assoziiert sind.

Für MigrantInnen, Personen in Haft und weitere Gruppen (z. B. SexarbeiterInnen) stehen aktuell keine Daten aus regelmäßigen Erhebungen zur Verfügung.

Aus den genannten Studien lassen sich neben dem Inputindikator zu den testenden Einrichtungen die Indikatoren zur Prävalenz, Impfquote und die Indikatoren der Behandlungskaskade für die jeweilige Gruppe erstellen (s. Online-Zusatzmaterial, Tab. Z-2).

Daten zum für Deutschland modifizierten Kernindikator „Anzahl an Einrichtungen, die eine Testung in Risikogruppen durchführen können“ (C.2), sind derzeit für MSM aus einem Teststellenprojekt des RKI verfügbar [45]. Eine Erfassung von niedrigschwelligen Drogenhilfeeinrichtungen mit Testangeboten für Drogengebrauchende ist in Planung durch das Zentrum für interdisziplinäre Suchtforschung, Universität Hamburg. Für andere Gruppen existieren derzeit keine geeigneten Datenquellen.

Für die Erhebung des „Anteils geheilter chronisch HCV-Infizierter mit abgeschlossener Behandlung“ (C.8.b) können sowohl Versorgungsdaten der GKV mittels Verknüpfung von Diagnose‑, Behandlungs- und ggf. Laborabrechnungsdaten verwendet werden als auch klinische Daten des Deutschen Hepatitis-C-Registers. Anhand letzterer Datenquelle oder alternativ mittels Apothekenabrechnungsdaten könnte der Indikator auch für PWID erhoben werden, da die Angabe zur Substitutionsbehandlung erfasst wird.

Die „Zahl der pro Jahr und Person abgegebenen sterilen Spritzen und Nadeln im Rahmen von Spritzentauschprogrammen“ (C.5) wird in einem Projekt der DBDD in Kooperation mit dem RKI und der DAH derzeit bestimmt und soll zukünftig regelmäßig erhoben werden. Die „Abdeckung der Opioidsubstitution bei PWID“ (A.24) wurde für das Jahr 2016 für Deutschland aus den Daten des Substitutionsregisters (Zähler; jährlich publiziert [46]) und der für 2016 publizierten Anzahl von Opioidabhängigen in Deutschland (Nenner) generiert [47]. MSM sind in Daten aus dem European MSM Internetsurvey (EMIS) abgebildet [48]. Da es sich um eine wiederkehrende Querschnittsbefragung handelt, stehen sowohl Daten aus der Basis- als auch aus den nachfolgenden Befragungen zur Verfügung.

Die Impactindikatoren zur Inzidenz C.9.a und C.9.b können anhand von DaTraV-Daten erhoben werden. Durch Hinzunahme von Abrechnungsdaten oder Diagnosedaten zur Opioidabhängigkeit kann der Indikator auch als Proxy für die PWID unter Substituierten/Opioidabhängigen erhoben werden. Alternativ kann der Indikator anhand der gemeldeten HBV-/HCV-Fälle pro Jahr geschätzt werden. Über die Angaben zu Geburtsland und Nationalität könnte der Indikator für die Subpopulation der MigrantInnen generiert werden.

## Diskussion

Durch die im RKI geleisteten Vorarbeiten und die Ergebnisse des Arbeitstreffens konnte eine Übersicht über die in Deutschland vorhandenen Datenquellen zur Hepatitis B und C erstellt werden. Daraus wurden konkrete Ideen und Handlungsaufgaben entwickelt, wie die von der WHO definierten und für Deutschland relevanten Indikatoren konstruiert werden können.

Alle identifizierten Datenquellen weisen spezifische Limitationen für ihre Verwendbarkeit auf. Für die Erhebung von Zähler und Nenner der Indikatoren müssen häufig verschiedene Datenquellen kombiniert werden. Im Folgenden werden die Besonderheiten der jeweiligen Datenquellen diskutiert.

### Daten aus Laboren

sind je nach Labor und dem jeweils angebotenen Diagnostikspektrum unterschiedlich verfügbar. Für die Bildung des Nenners wäre eine Sammlung der Daten von allen Laboren notwendig. Sollte nur ein Teil der Labore Daten liefern, könnten nur Positivenanteile benutzt werden. Daten zum HCV-Genotyp werden aufgrund des Einsatzes pangenotypischer Medikamente bei unkomplizierten HCV-Infektionen immer seltener nötig und daher nicht mehr routinemäßig erhältlich sein.

Da eine Verknüpfung mit Patienten- oder klinischen Daten nicht oder nur sehr eingeschränkt möglich ist, liegen den Laboren zum Teil keine Daten zum Stadium der Infektion vor. Testergebnisse aus unterschiedlichen Laboren zum gleichen Patienten, die z. B. bei Wiederholungs- oder Folgetestungen anfallen (z. B. erste Testung im ambulanten, allgemeinmedizinischen Bereich, zweite Testung im stationären Bereich oder bei Fachdisziplin) können bisher nicht verknüpft werden. Dadurch würden Schätzungen der Hepatitis-B- und -C-Prävalenz und -Inzidenz in Deutschland anhand von Labordaten vermutlich zu hoch ausfallen. Das könnte durch die Verknüpfung von Diagnose und Laborabrechnungsziffer in den KV- oder Krankenkassendaten vermieden werden. Mit Einführung des Deutschen elektronischen Meldesystems (DEMIS) kann jedoch die Nutzung von Labordaten für weitere Indikatoren neu evaluiert werden [49].

Unter der Annahme, dass bei der Diagnostik auf das Vorliegen einer viralen Hepatitis auch Labormarker erhoben werden, die die Leberfunktion abbilden, könnten Hepatitis-B/C-positive Testergebnisse mit dem APRI-Score verknüpft werden. Dadurch wären Labordaten für die Schätzung des Anteils HBV-/HCV-bedingter Leberzirrhosen nutzbar.

### Sekundärdaten,

die codierte Diagnosen sowie Abrechnungsziffern zu erfolgter Diagnostik oder Behandlung umfassen, stellen eine sehr umfangreiche Datenquelle dar, anhand derer viele Indikatoren erhoben werden könnten. Durch Kombination von Diagnose‑, Diagnostik- und Behandlungsabrechnungsziffern und Anwendung geeigneter Vorbeobachtungszeiträume können Inzidenzen und Prävalenzen sowie Co-Infektionen, Behandlungsbeginn, abgeschlossene Behandlungen, bei Erstdiagnose vorliegende Leberzirrhose oder HCC abgebildet werden. Mögliche Einschränkungen betreffen die Datenqualität. Die Daten bilden nur diagnostizierte oder in Behandlung befindliche Personen ab, deren Diagnose und Behandlung auch vom Arzt abgerechnet wurde. Damit ist die Datengüte abhängig von ärztlichem Codierverhalten und Versicherungsstatus. Vulnerable Gruppen lassen sich anhand von Abrechnungsdaten nicht valide identifizieren, so sind Inhaftierte oder auch Nichtversicherte in diesen Daten nicht abgebildet. Je nach Datenquelle ist die Verfügbarkeit der Sekundärdaten nur mit großem zeitlichen Aufwand gegeben und auch die Datenverwendung teilweise aufwendig und zeitintensiv. Allerdings könnten bei einmal etablierten Abfragen Folgeabfragen mit geringerem Aufwand durchgeführt werden. Für regelmäßige Abfragen und Verknüpfung unterschiedlicher Datenquellen sind gesetzliche Datenschutzvorgaben zu beachten.

Die Daten des Zi wären vorzugsweise für die Indikatoren „Testungen auf Hepatitis B“, „Testungen auf Hepatitis C“ sowie „HBsAg-Screening von Schwangeren“ zu verwenden. Letzterer Indikator wurde in der Vergangenheit bereits in einem Kooperationsprojekt von RKI und InGef erhoben [26]. Sekundärdaten zu Impfungen liegen in Form der RKI-Impf-Surveillance vor [23].

Die DaTraV-Daten enthalten Versorgungdaten aller GK-Versicherten und umfassen sowohl ambulante als auch stationäre Diagnosedaten sowie Angaben zu verordneten Arzneimitteln. Diese Datenquelle ist daher am besten geeignet, um die Indikatoren der Behandlungskaskade sowie die klinischen Indikatoren zu Spätfolgen abzubilden. Allerdings sind diese Daten nur mit aufwendigem Beantragungs- und Bearbeitungsprozess verfügbar.

Die Daten der deutschen Krebsregister werden für die Erhebung der HCC-Inzidenz und der Abschätzung der Mortalität an HBV/HCV-bedingtem HCC genutzt, liegen aber erst mit einem Zeitverzug von 2–3 Jahren vor. Neben diagnostizierten Fällen enthalten sie Informationen zum Krankheitsendpunkt. Daten zur Ursache des HCC werden derzeit noch nicht erhoben. Bisherige Berechnungen des Anteils von HBV/HCV-bedingtem HCC beruhen auf unterschiedlichen Datengrundlagen und weisen eine hohe Varianz auf. Die zukünftige Implementierung eines Leberkrebsmoduls, ähnlich bereits bestehender Module für die häufigsten Krebsarten, erscheint denkbar. Damit wäre auch die Erfassung von Grunderkrankungen möglich, alternativ durch Verknüpfung mit anderen Datenquellen, z. B. der Krankenhausdiagnosestatistik.

### Die Daten aus klinischen Registern oder Studien

sind insbesondere bei der Erhebung von Indikatoren hilfreich, die Behandlungsbedürftigkeit bzw. -erfolg einer HBV/HCV-Infektion evaluieren. Zu beachten ist, dass die klinischen Register nur die Gruppen abbilden, die im Register bzw. der Studie eingeschlossen wurden (z. B. Hepatitis-C-Register: nur diagnostizierte und therapierte HCV-Infizierte). Dadurch kann es zu einer Verzerrung der Ergebnisse kommen, die bei der Hochrechnung auf die Gesamtbevölkerung berücksichtigt werden muss.

Der Anteil geheilter chronisch HCV-Infizierter mit abgeschlossener Behandlung kann anhand der Daten des Hepatitis-C-Registers erhoben werden. Als Annäherung an den Indikator „Virussuppression bei behandelten chronisch HBV-Infizierten“ kann aus klinischen Daten der Anteil einer negativen Hepatitis-B-PCR (*Polymerase Chain Reaction*) bei HBsAg-positiven Personen gezogen werden. Der Anteil behandlungsbedürftiger HBsAg-positiver Personen und Hepatitis-D-Co-Infektionen bei chronischer HBV-Infektion könnte über Zentren der Deutschen Leberstiftung erhoben werden.

Bereits vorliegende *Daten aus Bevölkerungsstudien (DEGS, KiGGS)* sind aufgrund ihres großen Datenkörpers und der Repräsentativität der Daten für die deutsche Allgemeinbevölkerung am besten geeignet, um *Baseline*-Indikatoren zu Prävalenz und Impfquoten zu erheben. Zukünftig können die Daten für Erwachsene aus der bevölkerungsweiten gern-Studie des RKI generiert werden. Eine kontinuierliche Erfassung des HBV- und HCV-Status bei Blutspendern erfolgt durch die *Blutspender-Surveillance* nach Transfusionsgesetz. Diese Daten liegen dem RKI vor und können ebenfalls für die Prävalenz in der Gesamtbevölkerung genutzt werden, liefern allerdings aufgrund der Selektion von eher gesunden Personen lediglich einen unteren Schätzwert. Im RKI verfügbare Surveillance-Daten wie die *Meldungen nach IfSG* unterliegen einer gewissen Untererfassung, da sie von der Umsetzung der Meldepflicht durch die zur Meldung verpflichteten Akteure sowie von Teststrategien abhängig sind. Vulnerable Gruppen sind in den Meldedaten meist nicht gut abgebildet. Dennoch eignen sich diese Daten aufgrund ihrer Verfügbarkeit für die Konstruktion von Indikatoren zur Anzahl diagnostizierter HBV/HCV-Infektionen sowie zur Inzidenz.

Prävalenzdaten für die bzgl. Hepatitis B und C relevanten *vulnerablen Populationen* liegen aufgrund ihrer aufwendigen und zeitintensiven Erhebung in epidemiologischen Studien zeitlich verzögert und unregelmäßig vor. Für Vorjahre konnte zur Berichterstattung der *Baseline*-Indikatoren zum Teil auf publizierte Daten zurückgegriffen werden. Die fehlende Aktualität und schwierige Aktualisierbarkeit der Daten, die teilweise eingeschränkte Repräsentierbarkeit der Daten für die besonders von HBV/HCV betroffenen Bevölkerungsgruppen sowie teilweise fehlende Daten für z. B. Personen in Haft, SexarbeiterInnen oder bestimmte MigrantInnen-Gruppen schränken die Datenqualität und -verfügbarkeit ein [9]. Auch sind die wichtigen Indikatoren der Behandlungskaskade nicht für alle Gruppen gleichermaßen konstruierbar. Für Daten bei Drogengebrauchenden pilotiert das RKI seit April 2020 die Machbarkeit einer regelmäßigen Datenerhebung zu HBV/HCV bei NutzerInnen von niedrigschwelligen und Substitutions-Einrichtungen, die ab 2022 ausgerollt werden soll. Damit soll die regelmäßige Erhebung von Indikatoren zur Prävalenz, Impfabdeckung und Versorgungskaskade ermöglicht werden. Im Rahmen der geplanten gern-Studie werden repräsentative Basisdaten zu verschiedenen MigrantInnen-Gruppen erhoben, sodass erstmals eine Berichterstattung möglich wird.

## Schlussfolgerungen

Das Format des Arbeitstreffens war neuartig. Der direkte Austausch zwischen den unterschiedlichen Akteuren war für die Entwicklung neuer Erhebungsinstrumente zur Indikatorenerfassung ein wichtiger Beitrag. Alle ausgewählten Indikatoren wurden diskutiert und möglichen Datenquellen für die Erhebung zugeordnet. Nachdem die verschiedenen Optionen zur Erhebung im Rahmen des Treffens zusammengeführt wurden, war es Aufgabe der AutorInnen, die geeignetsten Datenquellen für die jeweiligen Indikatoren unter Beachtung der jeweiligen Limitationen zu identifizieren. Im nächsten Schritt sollen in Projekten mit Datenhaltern die jeweiligen Indikatoren generiert werden. Ziel ist es, kontinuierlich die Daten für eine regelmäßige Bestimmung der Indikatoren zu erheben, um die Umsetzung der Hepatitiseliminierungsstrategie zu verfolgen. Auf diese Weise können engmaschig Anpassungen im Gesundheitssystem vorgenommen werden, um das Ziel der Eliminierung von Hepatitis B und C bis zum Jahr 2030 zu erreichen.

## Supplementary Information



## Footnotes

1 Der Begriff „Sekundärdaten“ wird gleichbedeutend mit „Routinedaten“ verwendet: routinemäßig von der gesetzlichen Krankenversicherung (GKV) oder der Renten- und Unfallversicherung (Sozialdaten) erhobene Daten zu Abrechnungszwecken.
