# Supplementary material for: How to assess the elimination of viral hepatitis B, C, and D in Germany? Outcomes of an interdisciplinary workshop
Source: Bundesgesundheitsblatt Gesundheitsforschung Gesundheitsschutz. 2020 Dec 16;64(1):77–90. [Article in German] doi: 10.1007/s00103-020-03260-2 (PMC7772161; doi:10.1007/s00103-020-03260-2)
Supplement: Supplementary file 1 [file 103_2020_3260_MOESM1_ESM.pdf]

Bundesgesundheitsblatt 1/2021

Elektronisches Zusatzmaterial zum Beitrag:

Wie lässt sich die Eliminierung von Hepatitis B, C und D in Deutschland messen? Ergebnisse eines interdisziplinären Arbeitstreffens

Ruth Zimmermann*[1], Wiebe Külper-Schiek*[1, 2], Gyde Steffen[1], Sofie Gillesberg Lassen[1,3,4], Viviane Bremer[1], Sandra Dudareva[1] und die Hepatitis-Monitoring-Arbeitsgruppe

*Geteilte Erstautorinnenschaft

[1] Robert Koch-Institut, Abteilung für Infektionsepidemiologie, Fachgebiet 34 HIV/AIDS und andere sexuell oder durch Blut übertragene Infektionen, Berlin, Deutschland
[2] Robert Koch-Institut, Abteilung für Infektionsepidemiologie, Fachgebiet 33 Impfprävention, Berlin, Deutschland
[3] Robert Koch-Institut, Abteilung für Infektionsepidemiologie, Fachgebiet 35 Gastrointestinale Infektionen, Zoonosen und tropische Infektionen, Berlin, Deutschland
[4] Charité – Universitätsmedizin Berlin, Berlin, Deutschland

Korrespondenzadresse:
Dr. Ruth Zimmermann
Robert Koch-Institut, Abteilung für Infektionsepidemiologie,
Fachgebiet 34 HIV/AIDS und andere sexuell oder durch Blut übertragene Infektionen
Seestr.10
13353 Berlin
ZimmermannR@rki.de

Inhalt:

Tab. Z-1: Art, Limitationen und Besonderheiten verfügbarer Datenquellen zur Generierung der Indikatoren zum Monitoring von Hepatitis B, C und D in Deutschland
Tab. Z-2: Prioritäre Indikatoren und verfügbare Daten unterschiedlicher vulnerablen Bevölkerungsgruppen in Deutschland
Referenzen

Tab. Z-1: Art, Limitationen und Besonderheiten verfügbarer Datenquellen zur Generierung der Indikatoren zum Monitoring von Hepatitis B, C und D in Deutschland

	Datenquelle	Art der verfügbaren Daten	Besonderheiten und Limitationen	Quelle
Labordaten	**Daten aus Laboren**	• Anzahl durchgeführter Tests (HBV, HCV, HDV)<br>• Anzahl positiver und negativer Tests<br>• Daten zu Genotypisierungen (nur in einigen Laboren verfügbar)	• Datenverfügbarkeit und -umfang je nach Labor unterschiedlich<br>• Soziodemographische Daten (Geschlecht, Alter, Abrechnungsnummer) nur eingeschränkt verfügbar<br>• Klinische Angaben meist nicht vorliegend<br>• Keine Verknüpfung von Patientendaten auf Labordatenebene möglich, somit ggf. Überschätzung bei wiederholten Testungen in verschiedenen Laboren<br>• Wenn nur aggregierte Daten erhoben werden, müssen keine datenschutzrechtlichen Aspekte beachtet werden	[1]
Daten aus klinischen Zentren	**In der Deutschen Leberstiftung assoziierte Zentren**	• Klinische, virologische und Behandlungsdaten von PatientInnen, die in Zentren der Deutschen Leberstiftung behandelt werden	• Repräsentiert nur PatientInnen aus Zentren der Deutschen Leberstiftung<br>• Studien und aggregierte reguläre (z.B. jährliche) Datenerhebungen können mit geringem Aufwand initiiert werden	https://www.deutsche-leberstiftung.de/
	**Deutsches Hepatitis C-Register (DHC)**	• Klinische, virologische und Behandlungsdaten von >16.000 behandelten PatientInnen mit HCV-Infektion<br>• Daten zu vulnerablen Gruppen (z.B. Drogengebrauchende in Substitution, Genotyp)	• Dokumentation seit 11/2014; (retrospektive Daten zu Therapiebeginn ab 02/2014 verfügbar). Ab 01/2015 besteht eine Dokumentationsfrist von HCV-Fällen von 3 Monaten<br>• Nur Daten von behandelten PatientInnen (Einschlusskriterien > 18 Jahre, detektierbare Viruslast)<br>• Keine Screeningdaten<br>• Register wird von Deutscher Leberstiftung in Kooperation mit dem Berufsverband Niedergelassener Gastroenterologen Deutschlands geführt	http://www.deutsches-hepatitis-c-register.de/<br>[2]

Sekundär- und Registerdaten	**Destatis**<br>Daten des Statistischen Bundesamtes	*Primärstatistiken*<br>1. Krankenhausstatistik:<br>• Krankenhaus-Diagnosestatistik (Erfassung aller Behandlungsfälle im stationären Sektor (Vollerhebung)<br>• Fälle auf der Grundlage der ICD-Codes nach Alter und Geschlecht nach Behandlungsort, Wohnort<br><br>2. Todesursachenstatistik<br>• Medizinischer Teil der Todesbescheinigung<br>• Erfassung aller Sterbefälle der Personen mit Hauptwohnsitz in Deutschland (Vollerhebung)<br>• Grundleiden der verstorbenen Person mit ICD-Kode (=unikausale Todesursachenstatistik) nach Alter und Geschlecht;<br>• multikausale Daten der verstorbenen Person mit ICD-Codes (mittel-/langfristig)<br><br>*Weitere verfügbare Datenquellen*<br>• 3. Fragen zur Gesundheit im Mikrozensus<br>• 4. Orientierungswert für Krankenhäuser<br>• 5. Krankheitskostenrechnung<br><br>*Sekundärstatistiken*<br>• Fallpauschalen-bezogene Krankenhausstatistik bzw. Pauschalisierendes Entgeltsystem Psychiatrie und Psychosomatik (DRG-/PEPP-Statistik)<br>• Angaben zu Nebendiagnosen (Alter, Geschlecht) im Rahmen der DRG-Statistik<br>• Erfassung aller im Rahmen der DRG abgerechneten stationären Fälle in Deutschland (Vollerhebung durch Institut für das Entgeltsystem im Krankenhaus (InEK)<br>• Ambulante Diagnosestatistik aus der Risikostruktur-Ausgleichsverordnung (RSAV-Datensatz)	• Öffentlich zugängliche voraggregierte Daten mit Zeitverzug verfügbar - zu bestimmten Stichtagen des Folgejahres keine Nachfragen/Korrekturen möglich (Ausnahme: Todesursachenstatistik)<br>• Nur Erhebung dokumentierter Fälle<br>• Eine Einzelfall-Zusammenführung mit anderen Daten (bspw. Daten resultierend aus dem Infektionsschutzgesetz IfSG) ist nicht möglich	www.gbe-bund.de<br><br>https://www-genesis.destatis.de/genesis/online
	**DaTraV**	• Daten gemäß Datentransparenzverordnung vom	• Zeitverzug von derzeit 4 Jahren	https://www.bgbl.de/

	Daten des Informationssystem Versorgungsdaten des Bundesinstitut für Arzneimittel und Medizinprodukte (BfArM), ehemals des Deutschen Instituts für Medizinische Dokumentation und Information (DIMDI)[1]	10. September 20121<br>• Ambulante und stationäre Diagnosedaten (ICD)<br>• Arzneimittelverordnungen<br>• Detaillierte Darstellung nach Alter, Geschlecht und für ausgewählte Berichtsjahre nach Regionen	• Vollerfassung für GK-Versicherte (kein Selektionsbias)<br>• Keine Daten von PK-Versicherten<br>• Jahresübergreifendes Aufgreifen möglich<br>• Keine Leistungsdaten und Facharztkennung<br>• Aufwändiger Beantragungsprozess, Bearbeitungsdauer >1 Jahr<br>• aufwändiger Bearbeitungsprozess: für Abfrage muss Auswertungsschema oder eine lauffähige Syntax erstellt werden	xaver/bgbl/start.xav#__bgbl__%2F%2F*%5B%40attr_id%3D%27I_2012_42_inhaltsverz%27%5D__1597842333138
Sekundär- und Registerdaten	**Zi**<br>Zentralinstitut für die kassenärztliche Versorgung in Deutschland	*Vertragsärztliche Abrechnungs-Daten gemäß § 295 SGB V*<br>• Angaben zum Arzt / zur Praxis<br>• Angaben zum Versicherten<br>• Diagnosen (Arzt- und Patientenbezogen pro Quartal)<br>• Gebührenordnungspositionen Leistungsbedarf<br>• (Arzt und Patient/Quartal)<br><br>*Arzneimittelverordnungsdaten gemäß § 300 Abs.2 SGB V*<br>• Angaben zum Arzt / zur Praxis<br>• Angaben zum Versicherten<br>• Abgegebene Arzneimittel	• Zeitverzug von ca. 1-1,5 Jahren<br>• Vollerfassung für GK-Versicherte des ambulanten Sektors (kein Selektionsbias)<br>• Keine Daten von PK-Versicherten<br>• Keine Daten des stationären Sektors<br>• Für die Verknüpfung unterschiedlicher Datenquellen und bestimmte Abfragen ggf. Gesetzesgrundlage notwendig<br>• Etablierung von Abfragen sehr zeitintensiv, jedoch folgende reguläre Abfragen unkompliziert	https://www.zi.de/
	**InGef**<br>Institut für angewandte Gesundheitsforschung Berlin	• Ambulante und stationäre Abrechnungsdaten von 7,2 Mio. Versicherten (ca. 4 Mio. Versichertenanonyme/Jahr) aus 60 Krankenkassen (v.a. Betriebs- und Innungskrankenkassen),<br>• Daten zu Erkrankung, Verordnungen (Arzneimittel, inkl. Heil- und Hilfsmittel), Arbeitsunfähigkeits-Bescheinigungen	• Erfasst nur diagnostizierte Personen<br>• Zeitverzug der Übermittlung von ca. 3-9 Monaten<br>• Keine Rückschlüsse/Auswertungen zu einzelnen Krankenkassen oder Regionen (kleiner als Bundeslandebene)<br>• Verzerrung durch eingeschlossene Krankenkassen (z.B. weniger ländliche Wohnorte, mehr westdeutsche Versicherte als im gesamtdeutschen Vergleich)<br>• Open-Source-Datei verfügbar<br>• Analysen werden durch InGef durchgeführt	https://www.ingef.de/das-ingef/was-wir-machen/<br>[3]
	**WiDO**	• Krankenhausabrechnungsdaten (§ 301 SGB V, zuk.	• Verzerrung: nur AOK-Versicherte abgebildet	https://wido.de/filead

	Daten des Wissenschaftlichen Instituts der AOK	§ 303 SGB V/ § 21 Krankenhausentgeltgesetz), die ambulante Versorgung (§ 295 SGB V, zuk. § 303 SGB V), Arzneiverordnungen (§ 300 SGB V, zuk. § 303 SGB V) und Versichertenstammdaten von 24 Mio. AOK-Versicherten beinhalten	(tendenziell ältere Patienten)<br>• Erfasst nur diagnostizierte Personen<br>• Aggregierter Datensatz kann für eigenständige Analyse zur Verfügung gestellt werden	min/Dateien/Dokumente/Publikationen_Produkte/Buchreihen/Versorgungsreport/2015-2016/Kapitel%20mit%20Deckblatt/wido_vsr20152016_kap18.pdf
Sekundär- und Registerdaten	**WIP**<br>Daten des Wissenschaftlichen Instituts der PKV	• Arzneimittelabrechnungen (ATC-Code, Pharmazentralnummer) von ca. 70 % aller Privatversicherten	• Keine vollständige Abbildung der Arzneimittelversorgung (nur eingereichte Verordnungen)<br>• Zeitverzug zwischen Leistungsinanspruchnahme und Antrag der Kostenerstattung<br>• Kennziffer z.T. nicht so detailliert differenziert wie bei GKV-Daten	http://www.wip-pkv.de/startseite.html
	**ZfKD**<br>Zentrum für Krebsregisterdaten	• Fallbezogene Daten der Landeskrebsregister (seit ca. 2005 Daten für Gesamtdeutschland vorhanden) Patientencharakteristika<br>• Diagnose (ICD-10-Codes, Art der Diagnosesicherung, -zeitpunkt, Morphologie/ Lokalisation (ICD-O), Tumorstadium, Molekulardiagnostik*)<br>• Therapie (Behandlungsort* (Kreisebene))<br>• Ereignisse (Rezidiv, Metastase, Progression)<br>• Todesfall (Datum, Ursache)<br><br>* seit 2013 verfügbar	• Keine flächendeckende Erfassung → Schätzung<br>• Erhält nur Daten dokumentierter Fälle<br>• Keine regelhafte Übermittlung von Risikofaktoren oder Krankheitsursachen<br>• 2-Jahresverzug, aber kontinuierliche Übermittlung<br>• Ergänzungen der Daten im zeitlichen Verlauf möglich<br>• (zeitlich begrenzte) ergänzende „Übermittlungsmodule“ denkbar	https://www.krebsdaten.de/Krebs/DE/Content/ZfKD/zfkd_node.html
	**RKI-Impfsurveillance**	• Pseudonymisierte, ambulante Abrechnungsdaten gesetzlich Krankenversicherter zu Impfungen<br>• Daten aus allen 17 KVen Deutschlands (ca. 85 % der Bevölkerung in Deutschland)	• Quartalsweise Übermittlung der Daten von KVen<br>• Im RKI vorliegende Daten, viel Erfahrung durch Auswertungen zu anderen Impfungen<br>• Risikogruppen nur über Abrechnungsdaten abbildbar<br>• Zeitverzug vor allem bedingt durch IT- und Auswertkapazitäten	[4]

Daten aus epidemiologischen Studien und Routinesurveillance	**DEGS**<br>Deutsche Erwachsenen-Gesundheitsstudie<br>**gern-Studie**<br>Gesundheit und Ernährung in Deutschland	• Bevölkerungsrepräsentative deutschlandweite Studien bei Erwachsenen<br>• Deutsche Allgemeinbevölkerung (18-80 Jahre)<br>• DEGS 2008-2010<br>• gern-Studie 2021-2023<br>• Prävalenz für HBV- und HCV-Seromarker, HBV-Impfung aus Impfbüchern<br>• soziodemographische und Verhaltensmerkmale	• Gesundheitssurveys in Deutschland alle 10 Jahre<br>• Repräsentative Daten für Allgemeinbevölkerung<br>• Risikogruppen und MigrantInnengruppen nicht repräsentativ abgebildet<br>• Für die gern-Studie mit IMIRA sollen MigrantInnengruppen repräsentativ eingeschlossen werden	https://www.degs-studie.de/deutsch/home.html<br>https://www.gern-studie.de/<br>[5]
	**KiGGS-Studie**<br>Studie zur Gesundheit von Kindern und Jugendlichen in Deutschland	• Bevölkerungsrepräsentative deutschlandweite Studie bei Kindern und Jugendlichen<br>• Querschnitt und Längsschnitt<br>• KiGGS Basis: 2003-2006<br>• KiGGS Welle 2: 2014-2017<br>• Prävalenz für HBV, HBV-Impfung aus Impfbüchern<br>• soziodemographische und Verhaltensmerkmale	• Repräsentative Daten für die deutsche Allgemeinbevölkerung im Alter von 3-17 Jahren<br>• KiGGS Basis: Risikogruppen und MigrantInnengruppen nicht repräsentativ abgebildet<br>• KiGGS Welle 2: Stichprobe klein für Subgruppen-Analysen bei Markern mit niedriger Prävalenz, z.B. HBsAg und Anti-HBc	https://www.kiggs-studie.de/deutsch/home.html<br>[6]
	**IMIRA**<br>Improving Health Monitoring in Migrant Populations	• Im Rahmen der gern-Studie.<br>• Befragungsstichprobe von Personen mit nicht deutscher Nationalität im Rahmen der gern-Studie<br>• Ziel, repräsentative Daten für die Bevölkerung mit Migrationshintergrund zu erheben	• Weitreichende Maßnahmen zur Einbeziehung von Menschen mit Migrationshintergrund (Oversampling, übersetzte Anschreiben, Erklärfilme, Videodolmetschung)<br>• Verwendete Sprachen: Arabisch, Englisch, Italienisch, Kroatisch, Polnisch, Russisch, Türkisch	https://www.rki.de/DE/Content/Gesundheitsmonitoring/Themen/Migration/IMIRA/IMIRA_node.html
	**DRUCK-Studie**<br>Drogen und chronische Infektionskrankheiten<br><br>**DRUCK 2.0 Pilot**	• DRUCK-Studie: multizentrische Querschnittsstudie 2011-2014 zu Prävalenz von HBV, HCV, HIV, Wissen, Risiko- und Präventionsverhalten von Personen mit injizierendem Drogenkonsum in Deutschland (8 Städte)<br><br>• DRUCK 2.0 Pilot 2020-2022: zukünftiges Monitoringsystem zu Infektionen und Verhalten von Personen mit injizierendem Drogenkonsum in Deutschland	DRUCK-Studie:<br>• Responded driven sampling<br>• Daten nur eingeschränkt generalisierbar, da teils große Unterschiede zwischen den Studienstädten;<br>• beste zurzeit verfügbare Datenquelle für PWID in Deutschland<br>DRUCK 2.0 Pilot:<br>• Nach der Pilotphase 2020-22 Ausrollung und regelmäßige Datenerhebung geplant<br>• Convenience-Sample über heterogenes Sample von Einrichtungen	[7, 8, 9]
	**EMIS**<br>Europäischer MSM Internet Survey	• Internet-Befragungssurvey bei MSM zu sexuell übertragbaren Infektionen<br>• Bisher 2 Befragungen: EMIS 2010 und EMIS 2017<br>• Einschließlich HBV in 2010 und HBV/HCV in 2017	• Selbstberichtete Angaben zu HBV und HCV<br>• Convenience-Sample, aber sehr große Studienpopulation in Deutschland	[10, 11, 12]

Daten aus epidemiologischen Studien und Routinesurveillance	**HIV 1-Serokonverterstudie**	• Multizentrische, prospektive Langzeitkohorte (seit 1997)<br>• Kontinuierliche Sammlung von Blutproben und zugehörigen Fragebögen<br>• Serologische Daten zu HIV (Subtypen, rekombinante Formen) und HBV-/HCV-Ko-Infektionen sowie epidemiologische Daten von Personen mit bekanntem HIV-Infektionsdatum	• Studienkohorte (ca. 4.000 Teilnehmende; 86% MSM) gilt als repräsentativ für MSM in Großstädten<br>• Rekrutierung von Personen mit akuter HIV-Infektion bzw. mit bekanntem HIV-Infektionsdatum in teilnehmenden Studienzentren	[13, 14]
	**Daten der Blutspende-surveillance**	• Infektionen bei Blutspendern nach § 22 Transfusionsgesetz<br>• Meldungen aller Blutspendedienste zu HBV-, HCV-Infektionen bei Blutspendern (nach Alter und Geschlecht)	• Daten werden vom RKI erfasst und wissenschaftlich ausgewertet<br>• Veröffentlicht mit Zeitverzug von 1-2 Jahren<br>• Daten von Neuspendern dienen als unterer Schätzer für die HBV- und HCV- Prävalenz<br>• Inzidenz über die Jahre darstellbar	https://www.rki.de/DE/Content/Infekt/Blut/Transfusionsmedizin/Infektionsdaten/infektionsdaten_node.html<br>[15]
	**HBV- /HCV-Meldedaten nach IfSG**	• Meldedaten nach IfSG zu neu diagnostizierten HBV- und HCV-Infektionen nach Alter, Geschlecht und Landkreis<br>• Weitere Variablen vorhanden (z.B. Transmissionswege, klinische und Laborangaben, Stadium der Infektion, Nationalität, Geburtsland)	• Labor- und Arztmeldepflicht<br>• Daten im RKI vorhanden und zugänglich<br>• Veröffentlichung im Infektionsepidemiologischen Jahrbuch und als epidemiologischer Jahresbericht jeweils zu den Daten des Vorjahres<br>• Daten abhängig von Teststrategien, Meldeverhalten und Falldefinitionsänderungen	www.rki.de/hbv<br>www.rki.de/hcv<br>[16, 17, 18, 19, 20]
	**Schuleingangs-Untersuchungen**	• Angaben zu durchgeführten Impfungen bei Kindern (4-7 Jahre) aus den Impfpässen im Rahmen der Schuleingangsuntersuchung<br>• Vollständige und angefangene Impfschemata	• Jährliche Erhebung und Veröffentlichung<br>• Impfquoten werden nur für Kinder mit vorliegendem Impfpass berechnet, daher nur begrenzt übertragbar auf alle Kinder der Schuleingangsuntersuchungen<br>• mögliche Überschätzung von Impfquoten	[21, 22, 23]
	**OKaPII**<br>Online-Befragung von Krankenhaus-Personal zur Influenza-Impfung	• Daten von Krankenhauspersonal zum Impfverhalten, inkl. Impfstatus jeweils in aktueller und vorheriger Saison zu Influenza (seit 2017), Masern, (einmalig 2018), Hepatitis B (seit 2019)<br>• Soziodemographische Daten<br>• Daten zu Impfstatus (Grundimmunisierung, Auffrischimpfung durchgeführt?), Bestimmung von Anti-HBs und –Höhe erfolgt?	• Jährliche Durchführung; Befragung nach Influenzasaison<br>• Rekrutierung der Krankenhäuser über Deutsche Krankenhausgesellschaft (DKG) (E-Mail + Rundschreiben), zusätzlich Anschreiben privater Klinikverbände<br>• Online-Befragung von Krankenhaus-Personal<br>• Möglicher Selektionsbias aufgrund der Rekrutierungsform i.S. von besonders engagierten Krankenhäusern/Personal mit besonderem Interesse an der Influenza-Impfung	[24, 25]

[1] Die hier dargestellte Datenbasis bildet die Daten der Datentransparenzverordnung vom September 2012 ab. Dies war die Verordnung, die zum Zeitpunkt des Arbeitstreffens in Kraft war und auf die in den Kurzvorträgen Bezug genommen wurde. Am 10. Juli 2020 wurden Art und Umfang der zu übermittelnden Daten nach Datentransparenzverordnung in §§ 303a-e SGB V und ergänzend in § 303f neu definiert. Zeitgleich (Mai 2020) wurde die ehemalige Datenaufbereitungsstelle in ein neues Forschungsdatenzentrum mit Angliederung an das BfArM übergeben. Zu Besonderheiten bzw. ggf. bestehenden Limitationen beim Umgang mit den Daten nach DaTraV vom Juli 2020 liegen uns derzeit (Stand 20. August 2020) noch keine Erfahrungen vor.

Tab. Z-2: Prioritäre Indikatoren und verfügbare Daten unterschiedlicher vulnerablen Bevölkerungsgruppen in Deutschland

Vulnerable Population	Indikatoren, die für jede Population erhoben werden sollen	Zusätzliche populationsspezifische Indikatoren	Hauptdatenquellen für Erhebung der Indikatoren	Daten zur Populationsgröße
MigrantInnen	• C.1.a Prävalenz chronischer HBV-Infektionen<br>• C.1.b Prävalenz chronischer HCV-Infektionen<br>• C.3* Anteil geimpfter Personen in vulnerablen Gruppen<br>• C.6 HBV-/HCV- diagnostizierte Personen<br>• C.7.a Hepatitis B Behandlungsabdeckung<br>• C.7.b Hepatitis C Behandlungsbeginn<br>• C.8.a Virussuppression bei behandelten chronisch HBV-infizierten Patienten<br>• C.8.b Anteil geheilter chronisch HCV-Infizierter mit abgeschlossener Behandlung<br>• C.9.a* / C.9.b Rate an HBV-/HCV-Neuinfektionen<br>• C.10 HBV-/HCV-bedingte Todesfälle	• A.1 Hepatitis D-Ko-Infektionen bei Personen mit chronischer HBV-Infektion<br>• C.3.b Abdeckung der 3. HBV-Impfstoffdosis bei Kindern<br>• HBsAg-Screening bei Schwangeren<br>• C.3.a HBV-PEP bei Neugeborenen HBV-infizierter Mütter<br>• WHO Europa 0,5 % HBsAg- Prävalenz in geimpften Kohorten	• Zukünftig IMIRA (Improving Health Monitoring in Migrant Populations); im Rahmen der gern-Studie<br>• Schuleingangsuntersuchungen [21, 22]<br>• KiGGS [6], KiGGS Welle 2	Jährlich<br>Ausländerzentralregister<br>https://www.bva.bund.de/DE/Das-BVA/Aufgaben/A/Auslaenderzentralregister/azr_node.html
Personen, die sich Drogen injizieren (PWID)		• A.1 Hepatitis D-Ko-Infektionen bei Personen mit chronischer HBV-Infektion<br>• C.2* Anzahl an Einrichtungen, die eine Testung in vulnerablen Gruppen durchführen können<br>• C.5 Spritzen- und Nadeltauschprogramme<br>• A.24 Abdeckung der Opioidsubstitution	• DRUCK-Studie [7, 8, 9];<br>• zukünftig DRUCK-2.0<br>• Substitutionsregister [26];<br>• Projekt der Deutschen Beobachtungsstelle für Drogen und Drogensucht in Kooperation mit RKI und DAH;<br>• Daten aus Studie zu niedrigschwelligen (Test)Einrichtungen	2016<br>Opioidabhängige in Deutschland [27]
Personen in Haft		• C.2* Anzahl an Einrichtungen, die eine Testung in Risikogruppen durchführen können	-	Jährlich<br>https://de.statista.com/statistik/daten/studie/158317/umfrage/gefangene-und-verwahrte-in-deutschland-nach-art-des-vollzugs/
Männer, die Sex mit Männern haben (MSM)		• C.2* Anzahl an Einrichtungen, die eine Testung in Risikogruppen durchführen können	EMIS 2010, EMIS 2017 [12]	2016/17 [28]<br>2009 [29]

HIV-Koinfizierte		-	Für MSM: HIV-1-Serokonverterstudie [13, 30]	2018 https://www.rki.de/DE/Content/Infekt/EpidBull/Archiv/2019/Ausgaben/46_19.pdf
SexarbeiterInnen		• C.2* Anzahl an Einrichtungen, die eine Testung in Risikogruppen durchführen können	-	-

* Indikator für Deutschland adaptiert, C Kernindikator, A Zusatzindikator

Referenzen

1. Meurs L, Albrecht S, Bock Ct et al. (2020) Verfügbarkeit von virologischen und klinischen Informationen zu Hepatitis C in Routine-Laboren in Deutschland. Ergebnisse einer Laborbefragung zur Hepatitis-C-Diagnostik und -Meldung nach IfSG. Epid Bull 9:3-11.
2. Hueppe D, Serfert Y, Buggisch P et al. (2019) Deutsches Hepatitis C-Register (DHC-R) – eine Zwischenbilanz 4 Jahre nach Zulassung direkt antiviraler Substanzen (DAAs). Z Gastroenterol 57:27-36.
3. Andersohn F, Walker J (2016) Characteristics and external validity of the German Health Risk Institute (HRI) Database. Pharmacoepidemiology and drug safety 25:106-109.
4. Rieck T, Feig M, Eckmanns T, Benzler J, Siedler A, Wichmann O (2014) Vaccination coverage among children in Germany estimated by analysis of health insurance claims data. Hum Vaccin Immunother 10:476-484.
5. Poethko-Muller C, Zimmermann R, Hamouda O et al. (2013) Die Seroepidemiologie der Hepatitis A, B und C in Deutschland : Ergebnisse der Studie zur Gesundheit Erwachsener in Deutschland (DEGS1). Bundesgesundheitsblatt, Gesundheitsforschung, Gesundheitsschutz 56:707-715.
6. Cai W, Poethko-Muller C, Hamouda O, Radun D (2011) Hepatitis B virus infections among children and adolescents in Germany: migration background as a risk factor in a low seroprevalence population. Pediatr Infect Dis J 30:19-24.
7. Haussig JM, Nielsen S, Gassowski M et al. (2018) A large proportion of people who inject drugs are susceptible to hepatitis B: Results from a bio-behavioural study in eight German cities. International journal of infectious diseases : IJID : official publication of the International Society for Infectious Diseases 66:5-13.
8. Wenz B, Nielsen S, Gassowski M et al. (2016) High variability of HIV and HCV seroprevalence and risk behaviours among people who inject drugs: results from a cross-sectional study using respondent-driven sampling in eight German cities (2011–14). BMC Public Health 16:1-14.
9. Robert Koch-Institut (RKI) (2016) Abschlussbericht der Studie „Drogen und chronischen Infektionskrankheiten in Deutschland“ (DRUCK-Studie). In:Robert Koch Institut (RKI), Berlin http://www.rki.de/DE/Content/InfAZ/H/HIVAIDS/Studien/DRUCK-Studie/Abschlussbericht.pdf.
10. European Centre for Disease Prevention and Control (ECDC) (2013) The EMIS Network. EMIS 2010: The European Men-Who-Have-Sex-With-Men Internet Survey - Findings from 38 countries. In:ECDC, Stockholm http://ecdc.europa.eu/en/publications/Publications/EMIS-2010-european-men-who-have-sex-with-men-survey.pdf.
11. European Centre for Disease Prevention and Control (ECDC) (2019) EMIS-2017. The European Men-Who-Have-Sex-With-Men Internet Survey - Key findings from 50 countries. . In: ECDC Technical Report. European Centre for Disease Prevention and Control,, Stockholm, Sweden https://www.ecdc.europa.eu/sites/default/files/documents/European-MSM-internet-survey-2017-findings.pdf.
12. Brandl M, Schmidt A, Marcus U, An Der Heiden M, Dudareva S (2020) Are men who have sex with men in Europe protected from hepatitis B? Epidemiology and infection 148
13. Jansen K, Thamm M, Bock CT et al. (2015) High Prevalence and High Incidence of Coinfection with Hepatitis B, Hepatitis C, and Syphilis and Low Rate of Effective Vaccination against Hepatitis B in HIV-Positive Men Who Have Sex with Men with Known Date of HIV Seroconversion in Germany. PLoS One 10:e0142515.

14. Koppe U, Hanke K, Fiebig U et al. (2019) HIV-Studien und HIV-Projekte am Robert Koch-Institut. Epid Bull 49:527 – 539.
15. Offergeld R, Ritter S, Hamouda O (2012) HIV-, HCV-, HBV-und Syphilissurveillance unter Blutspendern in Deutschland 2008–2010. Bundesgesundheitsblatt-Gesundheitsforschung-Gesundheitsschutz 55:907-913.
16. Meurs L, Dudareva S, Diercke M, Altmann D, Bremer V, Zimmermann R (2019) Hepatitis-C-Meldedaten nach IfSG, 2016 – 2018: Auswirkungen der Änderungen von Falldefinition und Meldepflicht. Epidemiologisches Bulletin 30:275 – 228.
17. Zimmermann R, Meurs L, Schmidt D, Kollan C, Dudareva S, Bremer V (2018) Zur Situation bei wichtigen Infektionskrankheiten in Deutschland: Hepatitis C im Jahr 2017. Epid Bull:271-281.
18. Robert Koch Institut (RKI) (2019) Infektionsepidemiologisches Jahrbuch meldepflichtiger Krankheiten für 2018. In:Robert Koch-Institut (RKI), Berlin
19. Robert Koch-Institut (RKI) (2018) Infektionsepidemiologisches Jahrbuch meldepflichtiger Krankheiten für 2017. In:Robert Koch-Institut (RKI), Berlin
20. Robert Koch-Institut (RKI) (2017) Infektionsepidemiologisches Jahrbuch meldepflichtiger Krankheiten für 2016. In:Robert Koch-Institut, Berlin
21. Robert Koch-Institut (RKI) (2019) Impfquoten bei der Schuleingangsuntersuchung in Deutschland 2017. Epid Bull 18/2019:147-153.
22. Robert Koch-Institut (RKI) (2018) Impfquoten bei der Schuleingangsuntersuchung in Deutschland 2016. Epid Bull 16/2018:151 – 156.
23. Robert Koch-Institut (RKI) (2017) Impfquoten bei der Schuleingangsuntersuchung in Deutschland 2015. EpiBull 16/2017:137-142.
24. Neufeind J, Wenchel R, Bödeker B, O W (2018) OKaPII-Studie zur Influenza-Impfung: Impfquoten und Impfmotivation bei Klinikpersonal in der Influenza-Saison 2016/2017. Epid Bull. 10.17886/EpiBull-2018-040:313–321.
25. Boedeker B, Neufeind J, O W (2019) OKaPII: Influenza-Impfquoten-Monitoring im Krankenhaus. Epid Bull 10.25646/634:467 – 469.
26. Bundesopiumstelle Des Bundesinstituts Fuer Arzneimittel Und Medizinprodukte (Bfarm) (2020) Bericht zum Substitutionsregister 2020. In:Bundesinstitut für Arzneimittel und Medizinprodukte (BFArM),, Bonn, p 1-10. https://www.bfarm.de/SharedDocs/Downloads/DE/Bundesopiumstelle/SubstitReg/Subst_Bericht2020.pdf?__blob=publicationFile&v=2.
27. Kraus L, Seitz NN, Schulte B et al. (2019) Estimation of the Number of People With Opioid Addiction in Germany. Deutsches Arzteblatt international 116:137-143.
28. Scholz SM, Damm O, Elkenkamp S, Marcus U, Greiner W, Schmidt AJ (2019) Population size and self-reported characteristics and sexual preferences of men-who-have-sex-with-men (MSM) in Germany based on social network data. PLoS One 14:e0212175.
29. Marcus U, Hickson F, Weatherburn P, Schmidt AJ (2013) Estimating the size of the MSM populations for 38 European countries by calculating the survey-surveillance discrepancies (SSD) between self-reported new HIV diagnoses from the European MSM internet survey (EMIS) and surveillance-reported HIV diagnoses among MSM in 2009. BMC Public Health 13:919.
30. Schmidt D, Kollan C, Haußig J et al. (2017) Anhaltende Hepatitis-Ko-Infektionen und HBV-Impfbedarf bei HIV-infizierten Personen in Deutschland – Daten aus dem Hepatitis Screening in der HIV-1 Serokonverterstudie 2012 – 2016. In: DÖAK 2017. Salzburg
